# Understanding the PULSAR effect in combined radiotherapy and immunotherapy using transformer-based attention mechanisms

**DOI:** 10.3389/fonc.2024.1497351

**Published:** 2024-12-02

**Authors:** Hao Peng, Casey Moore, Debabrata Saha, Steve Jiang, Robert Timmerman

**Affiliations:** ^1^ Department of Radiation Oncology, University of Texas Southwestern Medical Center, Dallas, TX, United States; ^2^ Medical Artificial Intelligence and Automation Laboratory, University of Texas Southwestern Medical Center, Dallas, TX, United States; ^3^ Department of Immunology, University of Texas Southwestern Medical Center, Dallas, TX, United States

**Keywords:** AI, transformer, PULSAR, immunotherapy, radiotherapy

## Abstract

PULSAR (personalized, ultra-fractionated stereotactic adaptive radiotherapy) is the adaptation of stereotactic ablative radiotherapy towards personalized cancer management. It has potential to harness the synergy between radiation therapy and immunotherapy, such as immune checkpoint inhibitors to amplify the anti-tumor immune response. For the first time, we applied a transformer-based attention mechanism to investigate the underlying interactions between combined PULSAR and PD-L1 blockade immunotherapy, based on the preliminary experimental results of a murine cancer model (Lewis Lung Carcinoma, LLC). The radiation and administration of α-PD-L1 were viewed as two external stimulation signals occurring in a temporal sequence. Our study demonstrates the utility of a transformer model in 1) predicting tumor changes in response to specific treatment schemes, and 2) generating self-attention and cross-attention maps. The cross-attention maps serve as a biological representation of the semantic similarity between source and target sentences in neural translation, offering insights into the causal relationships of the PULSAR effect. Our model offers a unique perspective with the potential to enhance the understanding of the temporal dependencies of the PULSAR effect on time, dose, and T cell dynamics. In a broader context, our proposed framework offers the potential to explore varying intervals and doses for subsequent treatments while monitoring the biological parameters impacted by these perturbations. This approach can lead to more personalized and rational radiation or drug interactions.

## 1 Introduction

The field of combining radiotherapy and immunotherapy is rapidly evolving. The two can be synergized through various mechanisms, including immunogenic cell death, enhanced tumor antigen presentation, tumor microenvironment modulation, and abscopal effect (1–5). For example, radiation therapy can trigger cell death within tumor cells, which release damage-associated molecular patterns and tumor-specific antigens. These released molecules are to be recognized by antigen-presenting cells and act as signals to alert the immune system, initiating an adaptive immune response against the tumor. Concurrently, immune checkpoint inhibitors (ICI), can enhance this immune response by activating T cells to recognize and attack tumor cells presenting these antigens. One of the most well-known immune checkpoints targeted by these inhibitors is the programmed cell death protein 1 (PD-1) receptor and its ligands PD-L1 and PD-L2. By blocking the interaction between PD-1 and its ligands, ICI drugs such as α-PD-L1, prevent T cell exhaustion and enhance their ability to kill tumor cells.

Despite promising progress, combination of the two treatments has yielded lackluster results in both clinical and preclinical studies (6–15). One clinical trial studied the outcome of multisite, more conventionally delivered Stereotactic Body Radiation Therapy (SBRT) followed by pembrolizumab, reporting an overall response rate of 13.2% in advanced solid tumors (11). Another PEMBRO-RT phase 2 clinical trial in non-small cell lung cancer demonstrated a doubling in overall response when patients were treated with SBRT (3×8 Gy) followed by pembrolizumab (12). The synergistic impact has been extensively explored in preclinical models as well. One study delivered 10 to 24 Gy in 1 to 3 daily, or every other day fractions and began PD1/PD-L1 checkpoint blockade therapy within a day of radiation (10). Another preclinical study included 3 daily SBRT fractions of 8 Gy, followed by α-CTLA4 treatment, beginning on the day of the last fraction (13). Both studies demonstrated additive benefits when immune checkpoint inhibitor (ICI) blockade is given concomitantly with radiation. Moreover, there are conflicting reports regarding the optimal timing of PD-L1 therapy in relation to radiation (10, 13). While some studies showed no additive benefit when PD-L1 therapy was administered 6 days after a single dose of radiation (14), others suggested clear benefits of PD-L1 therapy given every 21 days after radiation (15). Defining the ideal combination continues to pose a considerable challenge.

Our team at UT Southwestern Medical Center (UTSW) is exploring PULSAR (personalized, ultra-fractionated stereotactic adaptive radiotherapy), which aims to deliver tumoricidal doses in a pulsed mode with long intervals (16). Longer intervals spanning weeks or months not only allow for greater recovery of normal tissue following an injury but may also help maximize potential synergies resulting from concomitant immune-oncology approaches. As adaptive immune response typically takes time to develop and reach its full effectiveness, exploring its temporal interaction with pulsed, more independent radiation doses (henceforth referred to as the PULSAR effect), presents an intriguing opportunity.

Optimizing treatment timing is crucial for maximizing the synergy between radiation and ICI therapy. Modeling synergy is challenging due to the complexities of both physical and biological processes involved. Recently, we used a recurrent neural network (RNN) to predict volume trajectories more accurately than conventional methods (17). Building on this, we propose employing a transformer model with attention mechanisms to further explore the temporal interactions between two treatment sequences. Inspired by neural machine translation, where a model translates sentences from one language to another, each “word” in our study corresponds to either radiation pulse or α-PD-L1 dose. Transformers are powerful and flexible architectures that have revolutionized natural language processing and beyond (18, 19). Their capability to handle long-range dependencies and parallelize tasks makes them suitable for processing sequential signals. In addition, the attention mechanisms enable the model to identify interactions between various parts of the sequence. We speculate that the cross-attention maps in our study may serve as a biological equivalent representation of the interaction, such as semantic similarity, observed between source and target sentences in neural translation.

In our study, the radiation and administration of α-PD-L1 are viewed as two external stimulation signals occurring in a temporal sequence. We developed the transformer model to accomplish two objectives: 1) predict tumor changes for a given treatment scheme, and 2) provide self-attention and cross-attention maps. From our perspective, we consider the second objective to be of greater significance than the first, as it has the potential to provide deeper insights into the causal relationships underlying the PULSAR effect, as well as more personalized and rational radiation or drug interactions.

## 2 Methods and materials

### 2.1 Problem formulation

Given the complexities of interactions involved in radioimmunotherapy, it is challenging to model the response of individual components such as tumor growth, radiation damage, T-cell infiltration, and signaling pathways. To understand this better, several characteristics of involved physical and biological processes are summarized below. For instance, tumor growth with neither radiation nor PD-L1 inhibitor follows an exponential model. The killing effect of a single radiation pulse is commonly modeled through a linear-quadratic (LQ) model or a universal survival model (20). After the radiation is delivered on a given day, the repopulation of tumor cells is time dependent and follows an exponential model, dependent on the half-time of repopulation (from less than a day to several weeks) and lag time (21). The interplay between radiation and checkpoint inhibitors such as PD-L1 antibody is even more complicated. On one hand, high dose radiation promotes both local and systemic immune responses and recruits CD8+ cytotoxic T cells to tumor sites by mechanism, such as the c-GAS-STING cytosolic DNA-sensing pathway (22). The recruitment process is time-dependent and takes up to several days. On the other hand, interferon gamma released by CD8+ T cells, leads to compensatory PD-L1 expression on tumor as a mechanism to prevent autoimmunity. Meanwhile, T_reg_ cells inhibit immune response by elevating the expression of CTLA4 to inhibit the activities of antigen-presenting cells, and releasing cytokines such as TGF-β and IL10 to suppress the functions of effector T cells (23–25). As a result, the net tumoricidal effect of combined therapy depends on radiation dose, α-PD-L1 dose, time, T cell dynamics and tumor microenvironment. Furthermore, as radiation pulses are applied and T cells are continuously recruited to tumor sites, radiations kill both tumor and T cells. However, there might be a difference between tumor-resident T cells and newly arrived T cells from lymph nodes in terms of radiosensitivity (26).

### 2.2 Small animal experiments

**Figure 1:**
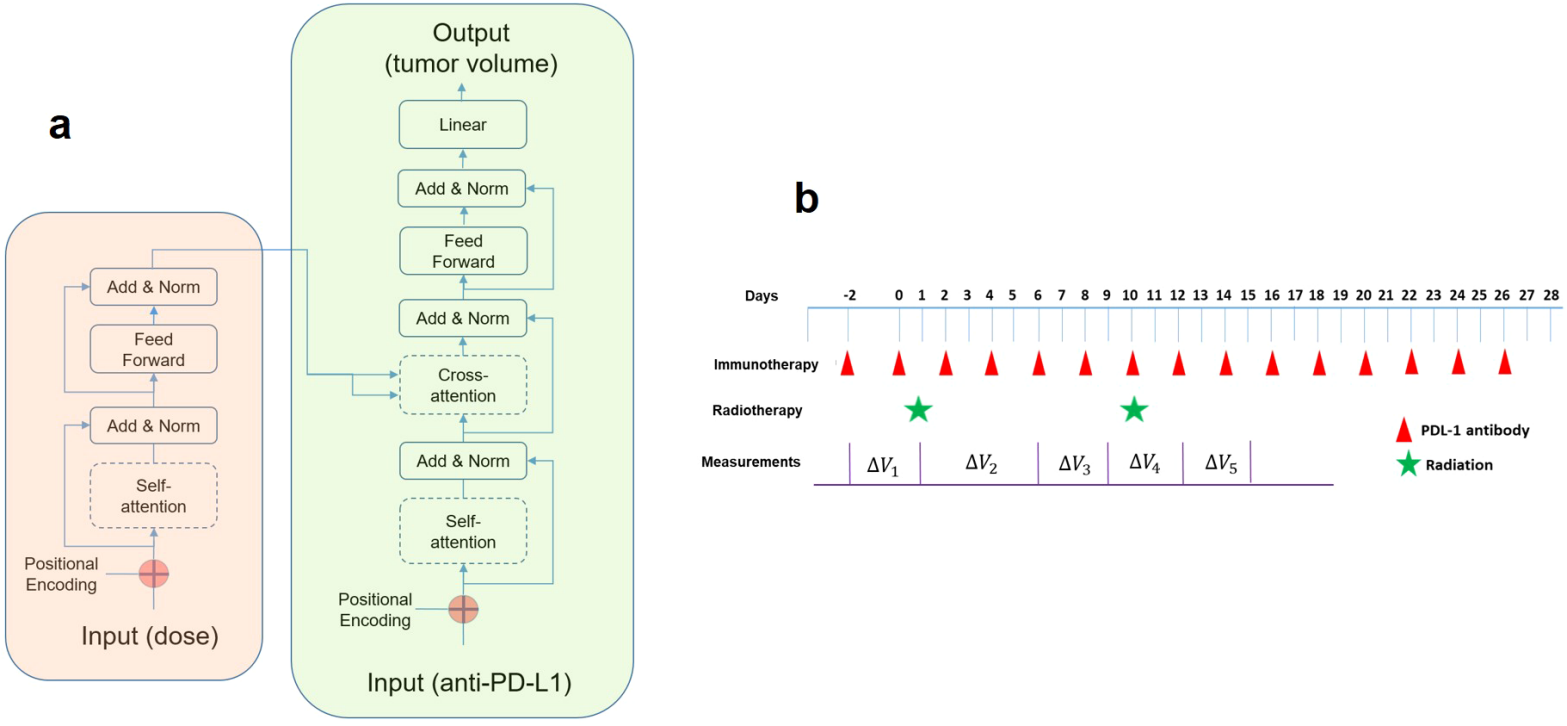
**(A)** The transformer model comprises an encoder (left box) and a decoder (right box). For a given external stimulus at a specific time, the attention score indicates the strength of its interaction to those inputs in the past. An attention score (also known as alignment scores, based on the query and key vectors) would stay between zero to one, and multiplying it with the value vector yields the context vector to be connected to the feedforward block for predicting tumor volume change. **(B)** The timing diagram of the combined therapy in our study. For immunotherapy, either α-PD-L1 or isotype control was administered. For radiation, different doses were delivered (10, 15, 20, 40 Gy). The first pulse of radiation was delivered 14 days after the implantation. Tumor volume measurements were conducted sequentially on selected days (not every day).

In this study, the impact of radiation schedules on tumor growth within an immune-resistant mouse model was studied (**Figure 1B**). Recognized as a “cold” tumor with low infiltration of T cells but high infiltration of myeloid-derived suppressor cells (MDSCs), Lewis lung carcinoma (LLC) offers valuable insights into the temporal behaviors of the adaptive immune response, as it takes a longer time to develop and reach its full effectiveness (27). In addition, differing from most preclinical models using radiation and with either daily or every other day fractions (10, 13–15), we tested radiation pulses with a longer spacing. The experimental protocols in this manuscript were approved by the Institutional Animal Care and Use Committee (IACUC) at UT Southwestern Medical Center (UTSW). The authors confirm that all animal procedures were performed in accordance with the animal experimental guidelines set by the IACUC at UTSW. UTSW uses the “Guide for the Care and Use of Laboratory Animals” when establishing animal research standards. The authors also confirm that the study was reported in accordance with ARRIVE guidelines (https://arriveguidelines.org). More details about the PULSAR study design can be found in our previous studies (16, 17). C57BL/6J mice were used, and LLC was derived from lung cancer of the C57BL/6 line. Tumor cells were injected subcutaneously on the right leg of mice. 10F.9G2 was used as an isotype control for α-PD-L1. Mice were administered (i.p) 200 μg α-PD-L1 or 200 μg isotype control according to different schedules (**Supplementary Table S1**). Local irradiations were conducted on a dedicated x-ray irradiator (X-RAD 32, Precision X-ray, Inc.) (**Supplementary Figure S1**). The tumor volumes were measured by length (x), width (y), and height (z), and calculated as tumor volume = xyz/2. If each length, width, or height of tumor was larger than 2 cm, the tumor volume was larger than 1500 mm^3^, or the mouse had significant ulceration in the tumor (see supporting materials), this indicated that the mouse reached the survival endpoint and so was euthanized by exposure to CO_2_. As a result, the data collection and volume measurement towards the end of the study showed an increased degree of variance, which was one of our study’s limitations. As noted in the study by Moore et al. (16), most experiments demonstrated no improvement in growth control with the addition of α-PD-L1 therapy compared to controls. This unexpected synergy pattern constitutes the PULSAR effect.

### 2.3 Data pre-processing

**Figure 2:**
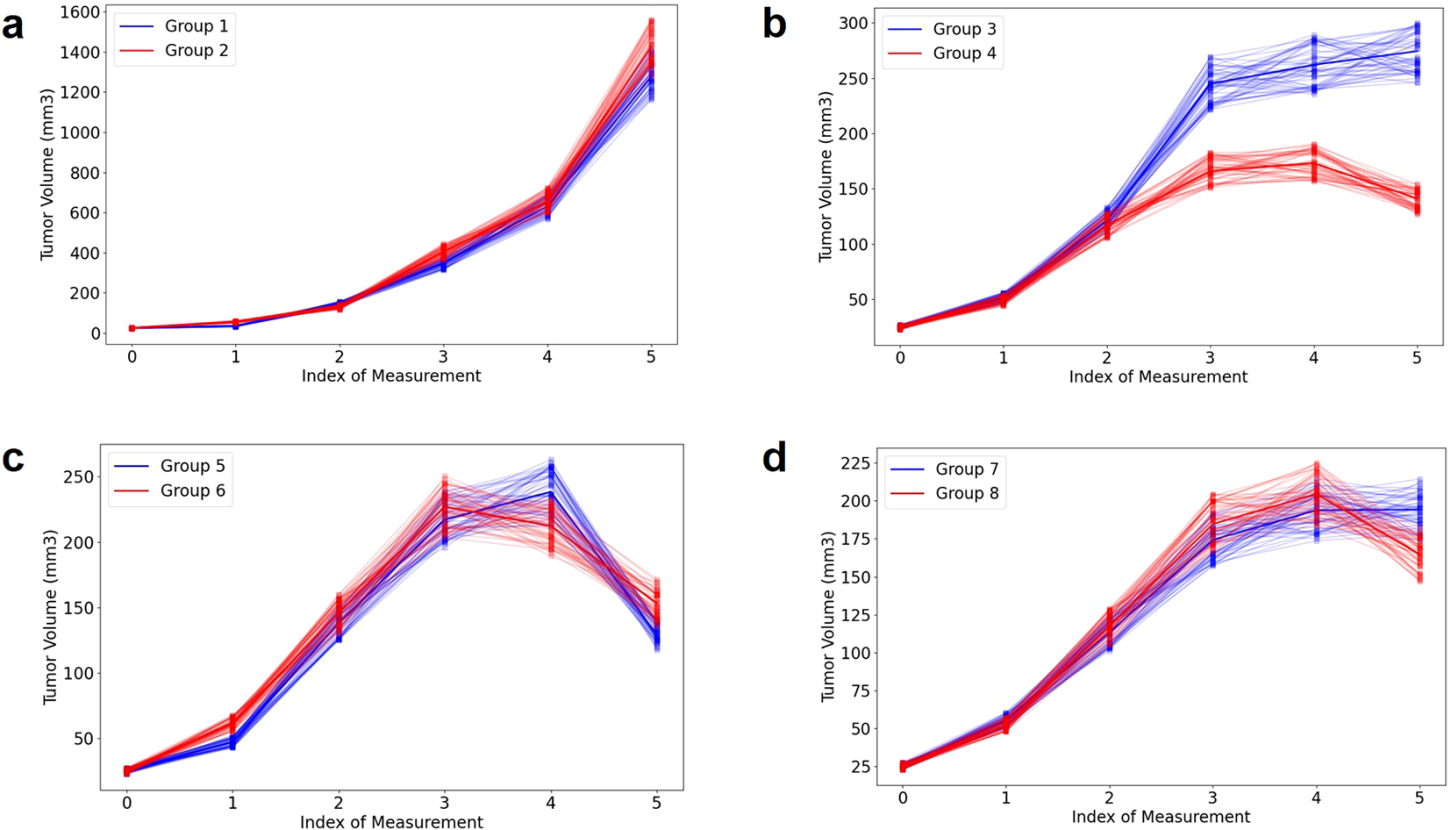
Illustration of tumor volume change under different delivery schemes for four pairs **(A–D)**. More details can be found in **Supplementary Table S1** and our previous publication (17). To increase sample diversity, each measurement’s distribution is represented by uniform sampling within ±10% of the mean.

For each treatment group, seven or eight animals were studied, and the measurement of tumor volume exhibited large variance. From a machine learning perspective, this weakens the one-to-one relationship between an input and output sequence. Consequently, data augmentation was necessary for enhancing sample diversity. The details of data processing and feature extraction are summarized below. Although the measurement was performed up to 40 days for some animals, an increased percentage of animals had missing data towards the survival endpoint due to severe ulceration. Therefore, the total time course was set to be 28 days (four weeks) for all samples to ensure data consistency. In total, we selected 24 treatment groups. For each group, the mean was computed at each measurement time point and fifty samples were randomly generated by uniformly sampling within a range of either 15% or 2% levels of the mean (e.g., two noise levels). For each group (with the same input), a higher noise level would lead to greater variance in the predicted output. As reflected in **Figure 2**, we acknowledge that synthesizing data in this manner does not address intra-animal variability and may obscure the overall trend. In this preliminary study, its primary purpose is to help prevent overfitting and improve generalization capability. 1200 samples were used for training (50 samples per group, 24 groups), and each sequence consisted of 28 steps (days), with two inputs and one output. The two inputs corresponded to radiation and α-PD-L1, respectively. The output sequence represented the tumor volume change, and if no input or output was available at a step, the values were set to zero.

### 2.4 Transformer model and attention mechanism

The concept was adopted from machine translation, where a model translates a sentence from a source language to a sentence in a target language. In simpler terms, the transformer model focuses attention on specific input words, and the attention acts as a link connecting the encoder and decoder. Each word in an input sentence is assigned its own query, key, and value vectors. These vectors are made by multiplying the encoder’s representation of the word with three distinct weight matrices developed during training. In this study, each word in a sentence corresponds to an input, representing either a radiation pulse or an α-PD-L1 dose. The execution of an attention layer involves five steps: 1) preparing hidden states and deriving a score for the encoder hidden state, 2) passing all scores through a softmax layer to generate attention distribution, 3) multiplying each encoder hidden state by its softmax score to acquire the alignment vector or annotation vector, 4) summing the alignment vectors to obtain the aggregated information from the previous step, and 5) inputting the context vector into the decoder. Due to the small size of the dataset, the transformer model was kept at a basic form with only 536 parameters. Every *“word”* represented a five-bit vector, with one bit indicating the physical input (based on the presence of α-PD-L1 or radiation dose) and four bits for positional embedding. A causal mask was applied to prevent the attention mechanism from sharing information about tokens at future positions (“don’t look ahead”), enforcing the autoregressive property during both training and inference. The decoder was constructed similarly, differing only in the output stage where a full connection was employed to predict tumor volume. We developed the transformer model using the Keras package (https://github.com/keras-team/keras).

To facilitate a better understanding of our approach, several points should be emphasized. Firstly, the transformer model focuses on the mean output of each group to identify differences in temporal response, neglecting inter-animal variation (e.g., noise level: 2%). Secondly, the tumor volume change at a specific time point is interconnected with all preceding inputs. The cumulative effect is implicitly considered by the transformer model. Thirdly, our current goal is to identify the PULSAR effect and potential synergy only in a qualitative manner. Quantitative analysis, if possible, needs to be postponed until additional biological correlates become available, such as the change of T cells. Fourthly, the attention maps in our model, reflecting the link between two treatment sequences, are more abstract compared to the semantics in neural translation.


(1) $$Attention\;Weigths=softmax\left(\frac{Q\;\cdot \;K^{T}}{\sqrt{d_{k}}}\right)$$

Volume Output =linear (Attention Weigths· V)

A plausible explanation of the link between the cross-attention map and the PULSAR effect is presented below. Inside the transformer model (**Figure 1A**), *Q* (queries), *K* (keys), and *V* (values) are vectors derived from the input data for calculating attention weights, as shown in Equation 1. *Q* represents the current token in the decoder’s input sequence for which we want to compute the attention weights. *K* represents all tokens in the in the encoder’s input sequence. V represents the actual values of the encoder’s input sequence to be used to generate the predicted output. The attention weight represents the importance of one token to another in a sequence, which is calculated using the dot product of the query (*Q*) and key (*K*) vectors, scaled by the square root of the key vectors’ dimension. In simple terms, it tells how much focus should be given to each token in the sequence. In machine translation, this reflects semantic similarity. In our study, it represents the temporal interaction between radiation and ICI drugs. These weights are then used to compute a weighted sum of the values *V* to get the final output, which is the volume change at selected time points in our investigation (**Figure 1B**). Both self-attention in the encoder (radiation) and cross-attention between the encoder and decoder were investigated. The latter helped in studying the relationship between radiation and immunotherapy concerning elapsed days.

### 2.5 Model training

The loss function was calculated based on the non-zero points (e.g. with measurements performed) only. The AdamW algorithm with default settings was used for optimization (learning rate = 0.0001, weight decay = 0.0001, beta1 = 0.9, beta2 = 0.999, epsilon = 1×10^−8^) (28). The batch size was 32 and the total epoch iteration was 5000. In total, 1200 samples (24 groups, 50 samples in each group) were generated from model training (80%) and testing (20%). To ease the learning task, the volume change between two adjacent measurement points was used as output. The L2 loss was used, representing the discrepancy between the predicted volume change (VC) (represented by Y in Equation 2) and experimentally measured VC. The VC difference (ΔVC) assumed either positive or negative values. If tumor volume was measured on 6 different days, we would have five ΔVC values. To assess the generalization capability and mitigate potential overfitting, holdout cross-validation was conducted. This involved testing the transformer model on 100 randomly sampled samples (out of 1200) that were not included in the training dataset. Note that summing five outputs would yield the total tumor volume at the endpoint (**Figure 1B**). However, assessing changes at multiple time points is preferable to a single-point assessment due to the additional information it provides. In other words, it allows us to identify the temporal evolution of synergy with greater granularity.


$$VC_{i}=Volume_{i+1}-Volume_{i}\;\;\;\;\;$$

(2) $${\text{ΔVC}}_{i}=Y_{i}-VC_{i}\;$$

## 3 Results

### 3.1 Prediction of tumor volume change

Here we briefly present four examples shown in **Figure 2** (noise level: 10%), to aid in understanding the AI modeling. In the absence of radiation (**Figure 2A**), there is no significant difference observed between the control group (group 1) and the α-PD-L1 antibody group (group 2). In the case of a single pulse of 20 Gy, there is a noticeable benefit in tumor control for group 4 (α-PD-L1) compared to group 3 (without α-PD-L1), which becomes more pronounced over time (**Figure 2B**). To clarify, the first measurement occurs three days before the initial radiation, the second on the day of the first radiation, and the third four days after the first radiation pulse. The overall trend in **Figure 2C** (a single pulse of 40 Gy, group 5: without α-PD-L1, group 6: with α-PD-L1) and **Figure 2D** (two pulses of 20 Gy with a 10-day interval, group 7: without α-PD-L1, group 8: with α-PD-L1) is like **Figure 2B**, while the PULSAR effect is observed toward the end of the trajectory and is less discernible due to the presence of large error bars. For additional cross-group comparisons in terms of PULSAR effect, please refer to our previous publication (17).

**Figure 3:**
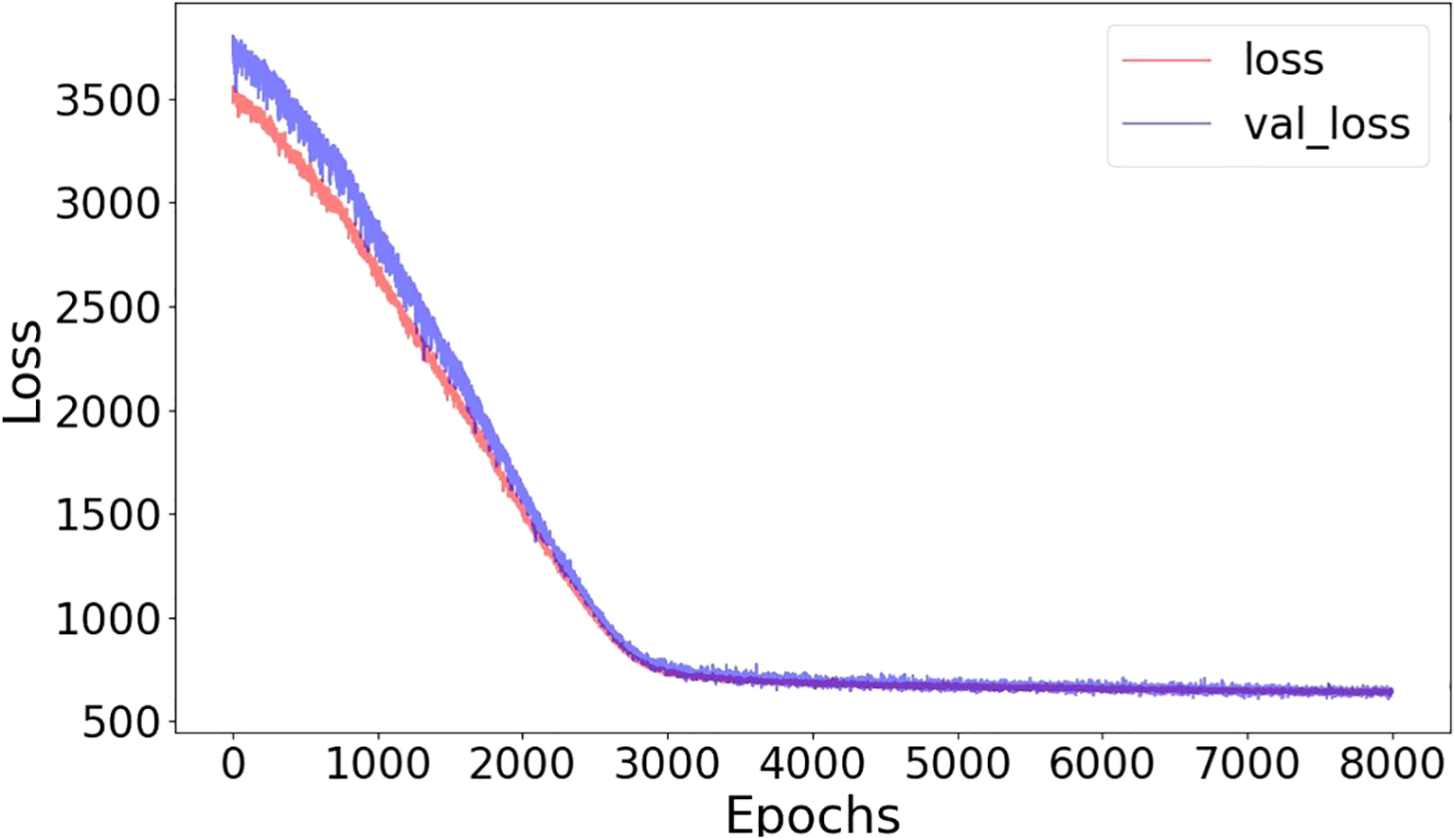
Training-loss and validation-loss curves in terms of mean-squared error as a function of epochs (batch size=32, noise level=10%).

**Figure 4:**
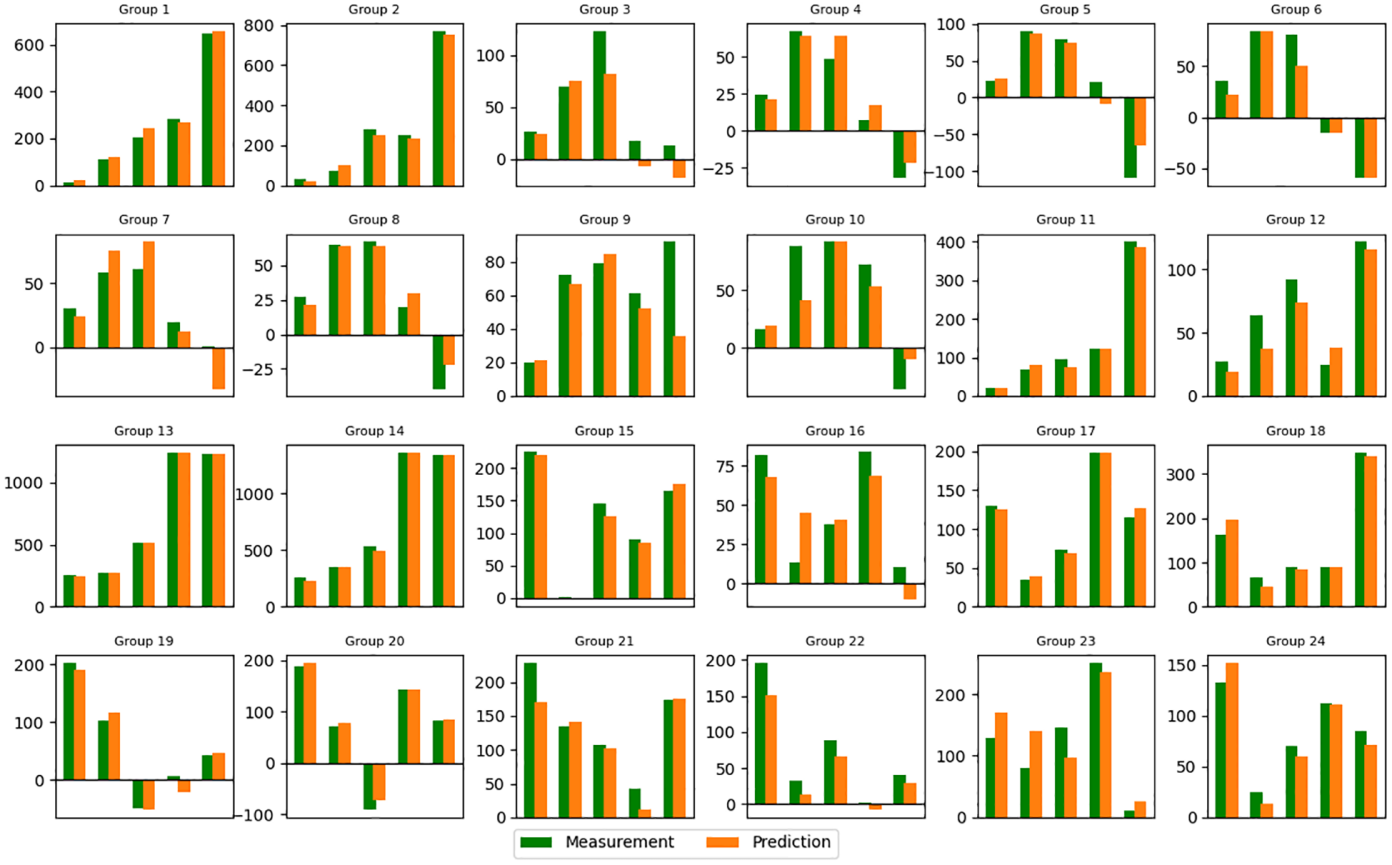
Predicted tumor volume change (mean value) for each group (noise level=2%). Refer to **Supplementary Table S1** in supporting materials for the detailed schedule of each group. Vertical axis: Volume change in cm^3^. Horizontal axis: Index of Measurement.

**Figure 5:**
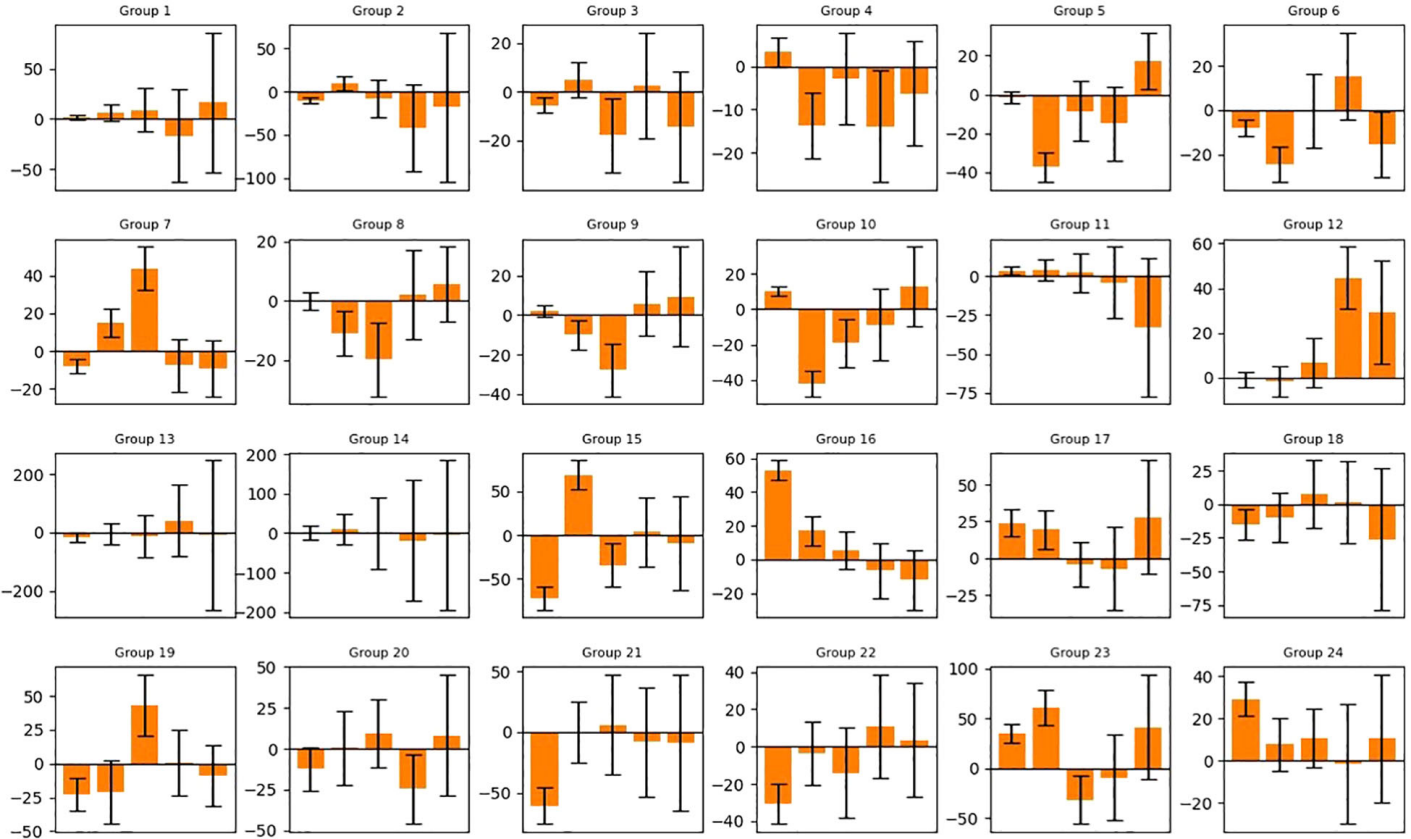
Predicted tumor volume change of 50 generated samples for each group (noise level=10%). The bars represent the average value and standard deviation of the prediction error. Refer to **Supplementary Table S1** for the detailed schedule of each group. Vertical axis: Volume change in cm^3^. Horizontal axis: Index of Measurement.

The training and validation loss converged after around 5000 epochs, showing no noticeable overfitting (**Figure 3**). In **Figures 4** and **5**, the prediction of tumor volume is presented for the noise level of 2% and 10%, respectively. In **Figure 4**, each bar represents the change between two adjacent measurements (only the mean is shown). For all groups, good agreement between predicted and experimental outcomes is observed. The discrepancy of a single bar falls within the range of -57 cm^3^ to 61 cm^3^ (e.g., the 5^th^ bar in group 9, the 1st bar in group 21, etc.).

In **Figure 5**, each bar represents the mean and standard deviation of the prediction error based on a randomly selected pool of 20 samples from 50. For most bars, the overall prediction accuracy remains within +/- 25 cm^3^ (e.g., good overall accuracy for each group), but a few exhibits large values over 50 cm^3^ (e.g., the 1st and 2nd bars in group 15, the 1st bar in group 16, etc.). The 10% noise level results in larger error bars and reduced accuracy compared to the 2% noise level, highlighting the adverse impact of data augmentation through uniform sampling for our task (i.e., one input, multiple outputs). From a machine learning perspective, the added noise obscures the overall trend of each volume trajectory and “confuses” the AI model. For this reason, we only selected the noise level of 2% for the subsequent analysis of the attention mechanism.

### 3.2 Self-attention maps

**Figure 6:**
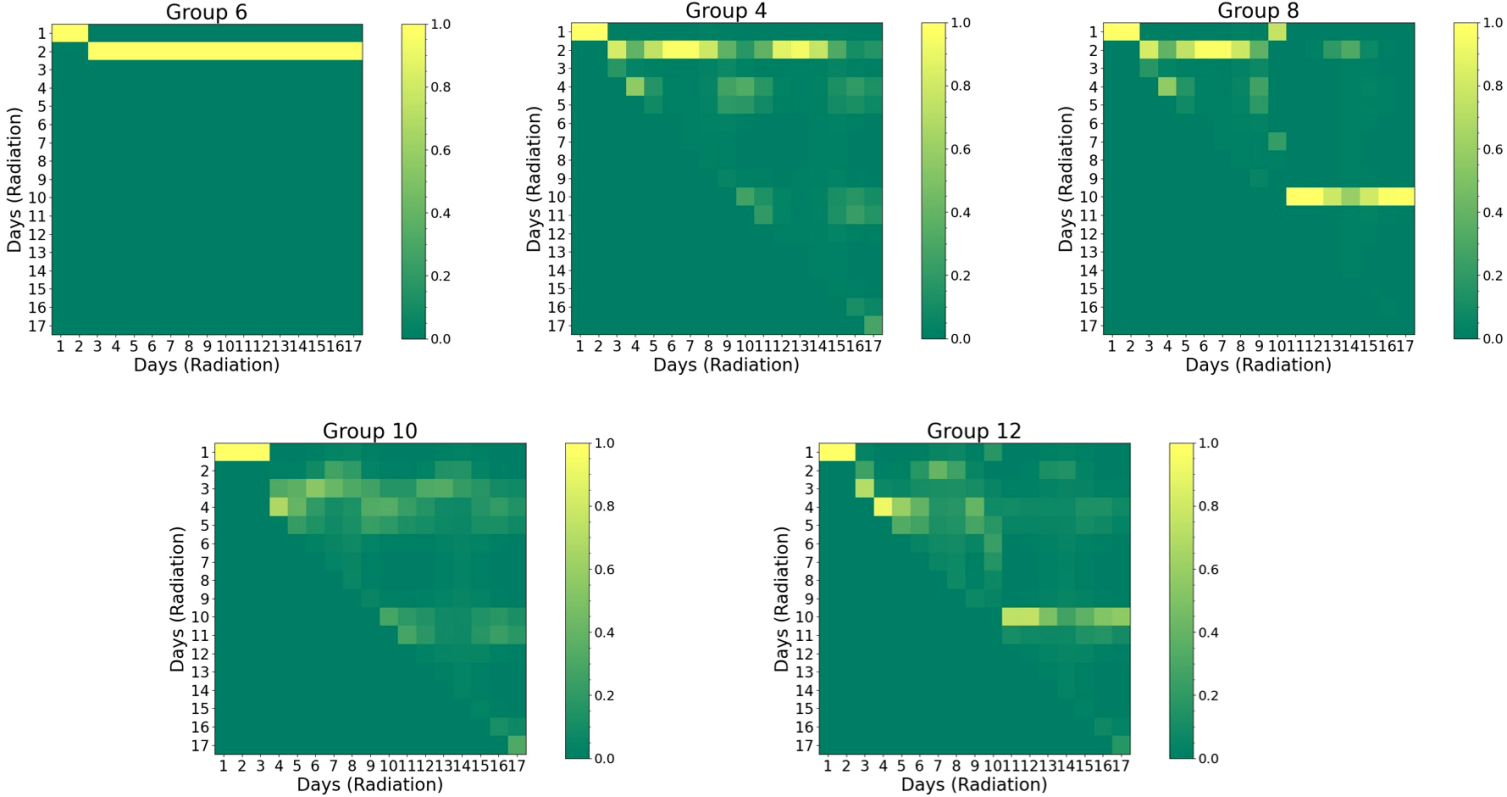
Visual representation of self-attention maps for selected groups group (noise level=2%). Both horizontal and vertical axes are associated with the sequence of radiation. The triangular pattern emerges from the application of a causal mask, which ensures that each location only has access to the locations that come before it. Pixels with higher values indicate greater attention and stronger interaction. Refer to **Supplementary Table S1** for the detailed schedule of each group.

**Figure 6** presents the self-attention maps (radiation vs. radiation). For both group 6 (“40Gyd1”, a single radiation of 40 Gy on day 1, with α-PD-L1) and group 4 (“20Gyd1”, with α-PD-L1), the impact of radiation is observable and the attention persists for an extended period of up to 20 days, with the former exhibiting a higher attention score due to a higher dose. The comparison between group 4 and group 8 (“20Gyd1 + 20Gyd10”, with α-PD-L1) shows that the second radiation pulse shifts the attention by 10 days and mitigates the attention score of the first radiation pulse after day 10. Similarly, between group 10 (“10Gyd1 + 10Gyd2”, with α-PD-L1) and group 12 (“10Gyd1 + 10Gyd10”, with α-PD-L1), the attention is shifted by 10 days when the two radiation pulses are spaced by 10 days. The cause of such an attention shift is not clear and may be attributed to the direct killing of both tumor cells and T cells by the second radiation, *“resetting”* the system to a new state.

### 3.3 Cross-attention maps

**Figure 7:**
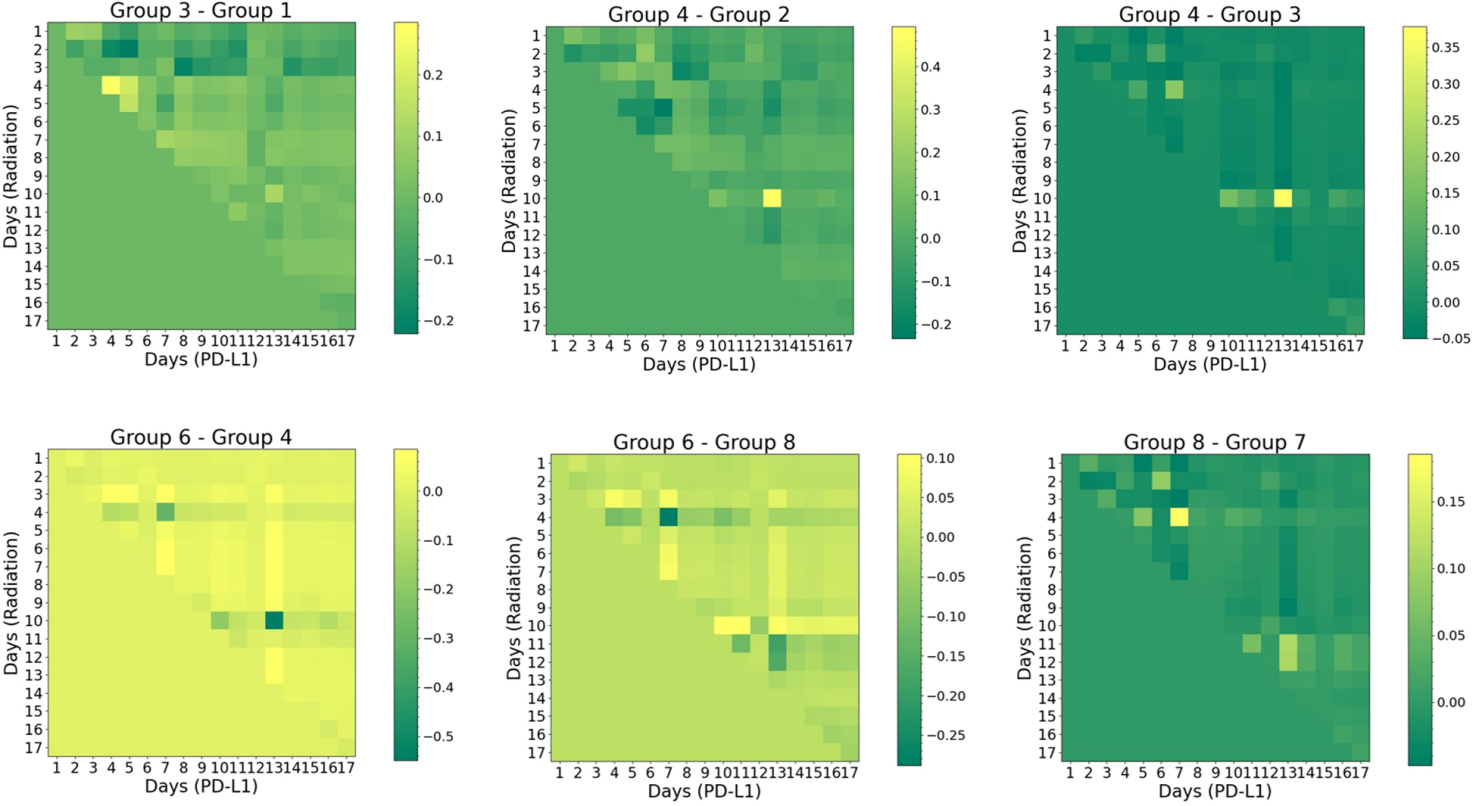
Visual representation of cross-attention maps for selected groups group (noise level=2%). The horizontal axis represents the sequence of α-PD-L1 sequence, while the vertical axis represents the sequence of radiation. The triangular pattern emerges from the application of a causal mask, which ensures that each location only has access to the locations that come before it. Refer to **Supplementary Table S1** for the detailed schedule of each group.

In **Figure 7**, the cross-attention plots reveal the temporal dynamics of the interaction between two treatments (radiation vs. α-PD-L1). To understand the respective contributions, the difference map between the two groups identifies the exclusive effect of either radiation or α-PD-L1 through straightforward arithmetic subtraction. For instance, both group 1 (no radiation) and group 3 (“20Gyd1”), were administered the isotype control, but only group 3 experienced a single 20 Gy radiation pulse on day 1. The difference in the attention score (“Group 3- Group 1”) is thus solely attributed to radiation, which peaks on the 4^th^ row and diminishes gradually over time. It is important to note that the relationship is not one-to-one, but rather one-to-many, indicating that the synergy (in the context of tumor volume change as output) persists for a finite period, gradually diminishing in strength.

Another interesting pattern arises in the comparison between group 4 (“20Gyd1”, matching group 3 but with α-PD-L1) and group 2 (no radiation, matching group 1 but with α-PD-L1). The impact of radiation is found to peak on the 10^th^ row and the 13^th^ column, where the maximum synergy occurs. Such a “lagging” pattern also emerges in the comparison between group 4 and group 3, where the difference is ascribed solely to α-PD-L1 with the presence of a single radiation dose at the onset. These results imply that the PULSAR effect is not instantaneous but takes time to build up.

Three additional vignettes below further illustrate how the PULSAR effect depends on radiation dose and scheduling. Between group 6 (“40 Gyd1”, with α-PD-L1) and group 4 (“20 Gyd1”, with α-PD-L1), the former one displays higher attention (positive difference) in most pixels, except for a few pixels where there is a negative difference (e.g., on the 4^th^ and 10^th^ rows). This suggests that although a dose of 40 Gy has a more pronounced killing effect, it may not necessarily lead to optimal synergy at other time points.

In the comparison between group 6 (“40 Gyd1”, with α-PD-L1) and group 8 (“20Gyd1 + 20Gyd10”, with α-PD-L1), the left half of the attention map exhibits the same pattern as in Group 6 - Group 4 (note the different scale of the color bar). One pattern emerges in the region corresponding to the second 20 Gy pulse (day 10 on the axis of radiation): the attention score flips its sign for multiple pixels, indicative of the change of attention (interaction) triggered by the second pulse of 20 Gy. Another comparison is shown between group 7 (“20Gyd1 + 20Gyd10”, without α-PD-L1) and group 8 (“20Gyd1 + 20Gyd10”, with α-PD-L1), illustrating the dependence of interaction and PULSAR effect on whether α-PD-L1 is used. It differs from the pattern of “Group 4-Group 3” (note the different scale of the color bar) after 10 days, due to the second 20 Gy pulse.

## 4 Discussion

Modeling interaction and synergy between concurrent radiation and ICI therapy is a challenging task. In this preliminary study, we attempted to tackle that challenge by employing an AI approach. The transformer model, coupled with the attention mechanism, leverages its unique capacity to process sequential information and capture underlying correlations (18, 19). As illustrated in **Figures 4** and **5**, it can predict the overall trend of tumor volume change for up to three weeks. The patterns depicted in **Figures 6**, **7** further shed light on the temporal interplay between the two. We would like to emphasize that for the time being, many of our conclusions are highly speculative and we do not yet fully understand the interpretation of attention maps. Our work lays the foundation for exploring the PULSAR effect, however at this point several interpretations are speculative and further research with more comprehensive data is needed.

Three important implications related to the use of a transformer in Equation 1 should be highlighted. Firstly, the analysis of cross-attention represents the temporal evolution of interaction and synergy, reflecting the lasting effects once a treatment is administered. For instance, if radiation is delivered on day 1, its impact would persist over a finite period, rather than being instantaneous. Secondly, the attention map does not indicate a direct correlation with volume change (e.g., not including V in Equation 1), and the analysis of attention remains only qualitative rather than quantitative. Thirdly, a novel aspect of our study is that by maintaining consistency among other variables and altering one variable at a time through arithmetic operation, such as comparing with or without radiation, or with or without PD-L1, changes in attention can be attributed to radiation and α-PD-L1 separately. As a result, this proposed framework can enable us to explore how the PULSAR effect shifts when radiation is administered at precise time points.

Ordinary Differential Equation (ODE)-based mathematical models have been extensively used for analyzing complex biological systems, such as utilizing discrete-time equations to model tumor control under radiation and the interplay between immunotherapy and radiotherapy (29, 30). The two may complement each other and enhance their interpretation. In our view, the transformer model offers several distinct advantages for investigating the PULSAR effect, particularly in terms of “causal relationship.” To elaborate, predicting the tumor volume trajectory is a curve fitting task. With a limited number of parameters and compartment models, an ODE-based approach may not be able to achieve good fitting results, given the complexity of the biological processes involved in combined RT and ICI treatment. Moreover, although other AI models such as RNN can identify the underlying correlation in sequential signals and predict volume change (17), RNN cannot provide information about temporal interaction. One unique advantage of the transformer is that the arithmetic operation in attention maps helps pinpoint the respective contribution from each treatment as a function of days. When a more continuous measurement of tumor volume or biological markers (e.g., cytotoxic T cells and regulatory T cells), we expect the cross-attention maps like **Figure 7** to become smoother and easier to interpret. An even broader question arises regarding the level of personalization in combined PULSAR and immunotherapy. Eventually, by leveraging the temporal interactions at different time points for each animal, we may identify optimal target-specific treatment strategies through reinforcement learning, as demonstrated in previous studies (31, 32).

Whatever modeling tools selected, we posit that a deeper understanding of synergy in concurrent RT and ICI therapy can be gained through the examination of the PULSAR effect, an immunomodulatory impact that can be either inhibitory, stimulatory, or neutral. From a mechanistic perspective, it helps gauge the binding between PD-1 and PD-L1 in our study, contingent on multiple factors such as timing, dose, tumor microenvironment, and T cell dynamics. The practical application of our proposed framework is two-fold.

On one hand, it can be used as an *in-silico* tool to explore new combinations that maximize synergy and therapeutic efficacy, out of many treatment permutations. A PULSAR trial including concomitant ICI blockade can thus be designed and conducted more cost-effectively. It may also help interpret result discrepancy in published studies examining the synergy between radiation therapy (whether daily fractionation or PULSAR) and immunotherapy regarding timing and dose (6–15). If the interval between two pulses is too brief, the second radiation may eliminate newly recruited T cells entering the tumor microenvironment. Conversely, excessively long intervals may hinder the optimal realization of therapeutic benefits from each treatment. Overly close intervals between the two pulses may even stimulate tumor growth. For instance, a study using similar preclinical models demonstrated accelerated tumor growth and decreased survival when 3 Gy doses were administered daily (33, 34). Likewise, the timing of administering α-PD-L1 needs to be optimized, given the time-dependence of the PULSAR effect and tumor microenvironment. In our previous study (16), the “cold” LLC tumor model exhibited the maximum synergy when radiation pulses were spaced 10 days apart, concurrently with the administration of the second α-PD-L1 dose. By contrast, “hot” tumor models like colon carcinoma (MC38 cells) exhibited maximum synergy immediately after the first radiation dose due to pre-existing immunity (16, 35).

On the other hand, it can be combined with biomarker identification to better investigate causal relationships relevant to the PULSAR effect. In the next phase of our study, we plan to integrate information on two aspects: 1) attention maps that identify the influence of each treatment, and 2) experimental measurements of biomarkers related to adaptive immune response (e.g. CD8+ T cells and T_reg_ cells). By overlaying these data along the same time axis, we can better understand causal relationships and rethink answers to several pertinent questions. For example, is the PULSAR effect instantaneous, or does it take time to build up? How does the first radiation pulse recruit CD8+ T cells over time? Does the second radiation pulse kill both tumor cells and T cells, “resetting” the system to a new state? How does each treatment alter the number and function of CD8+ T cells, T_reg_ cells, and other lymphocytes? For instance, one study reported that T cells (mostly T_reg_ cells) residing in the tumor during radiation display increased resistance to radiation, playing a critical role in overall tumor control (26). By contrast, T cells in other body compartments exhibit less resistance to radiation. Although the authors did not administer a second dose of radiation after the initial one, it raises the possibility that a subpopulation of newly recruited T cells may infiltrate and develop into intratumoral T cells of different radiosensitivity. If that is the case, maximizing the PULSAR effect may demand a boost of either radiation dose or α-PD-L1 dose when repetitive radiation is applied. In addition, several *in-vitro* studies have demonstrated the combined effect of T_reg_ regulation and radiation therapy (23–25). In a study involving mice irradiated with 10 Gy to the right leg (prostate C1 cells) (25), T_reg_ cells significantly increased in the spleen, lymph nodes, blood, and lung within two days after exposure, returning to normal levels by 10 days. Conjoining the temporal behavior of these biomarkers with the AI modeling will further validate the interpretation of the attention mechanisms.

The tasks outlined in this manuscript mark merely the beginning, with numerous limitations requiring further investigation. Firstly, the role of transformer-based AI modeling necessitates scrutiny, and the interpretation of attention mechanism needs to be validated. Secondly, the small number of groups results in insufficient sample diversity. The impact of data augmentation, either through uniform sampling or other methods, on the model’s performance remains a topic for future exploration. Our current goal is to discern the overall trend of each volume trajectory. The current approach with uniform sampling was not statistically rigorous and has inadvertently introduced some spurious noise (e.g., 10% noise level). Thirdly, the large error bars depicted in **Figure 2** indicate significant variability in measured tumor volumes within each group, resulting from both differing responses of animals and human measurement errors. To enhance precision in volume measurements, future studies should consider employing advanced contouring techniques using cone beam CT instead of manual measurements. Fourthly, the presence of numerous zeros in the output sequence is due to tumor volumes not being measured on multiple days. A continuous measurement of tumor volume would be more advantageous.

## 5 Conclusion

Combined radiotherapy and immunotherapy are a vast and intricate field, and AI represents just a small piece of the puzzle in unraveling its complexities. We developed a transformer-based AI model to study the temporal interaction between the two. While the synergy between them is supported by a growing body of evidence, this combination is not universally effective for all patients or all types of cancer. PULSAR presents a unique opportunity to personalize treatment and harness synergy more effectively. Our work has potential to enhance our understanding of how the PULSAR effect depends on time, dose and T cell dynamics. It can be used for in-silico modeling, facilitating the exploration of innovative treatment permutations. In a broader context, our proposed framework allows for the exploration of various intervals and doses for subsequent treatments while tracking the biological parameters influenced by these changes. This approach could result in more personalized and rational strategies for radiation or drug interactions and support the development of digital twins for cancer treatment.

## Data Availability

The raw data supporting the conclusions of this article will be made available by the authors, without undue reservation.
