# Supplementary material for: Understanding the PULSAR effect in combined radiotherapy and immunotherapy using transformer-based attention mechanisms
Source: Front Oncol. 2024 Dec 2;14:1497351. doi: 10.3389/fonc.2024.1497351 (PMC11647037; doi:10.3389/fonc.2024.1497351)

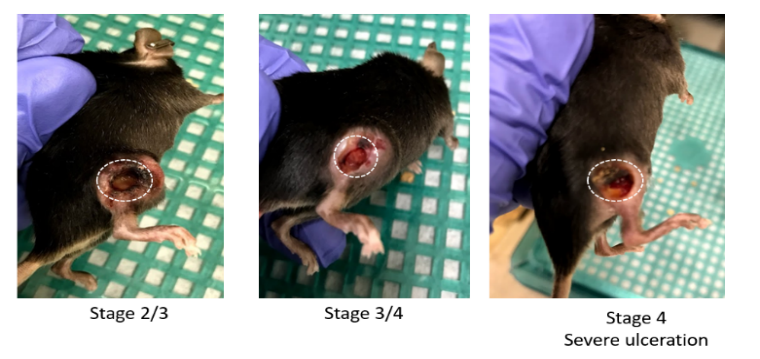

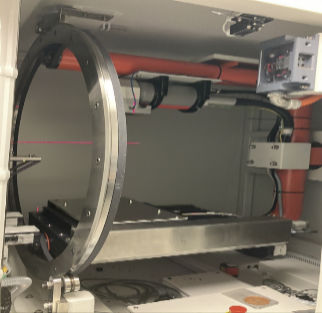


**Fig. S1.** (**left**) Experiential set up. Tumor-bearing mice were irradiated with 10 to 40 Gy according to multiple schedules (**Table S1**), using local irradiations with a small animal dedicated x-ray irradiator (X-RAD 32, Precision X-ray, Inc). (**right**) The tumor volumes were measured by length (x), width (y), and height (z) and calculated as xyz/2. If the tumor volume exceeds 1500 mm^3^, or the mouse has significant ulceration in the tumor, the mouse reaches the survival endpoint and is euthanized.

**Table S1.** The timing diagram of the combined therapy in our study (24 groups in total). For immunotherapy, either anti-PD-L1 or isotype control was administered. For radiation, different doses were delivered (10, 15, 20, 40 Gy). The first pulse of radiation was always delivered 14 days after the implantation. Tumor volume measurements were carried out sequentially on certain days.


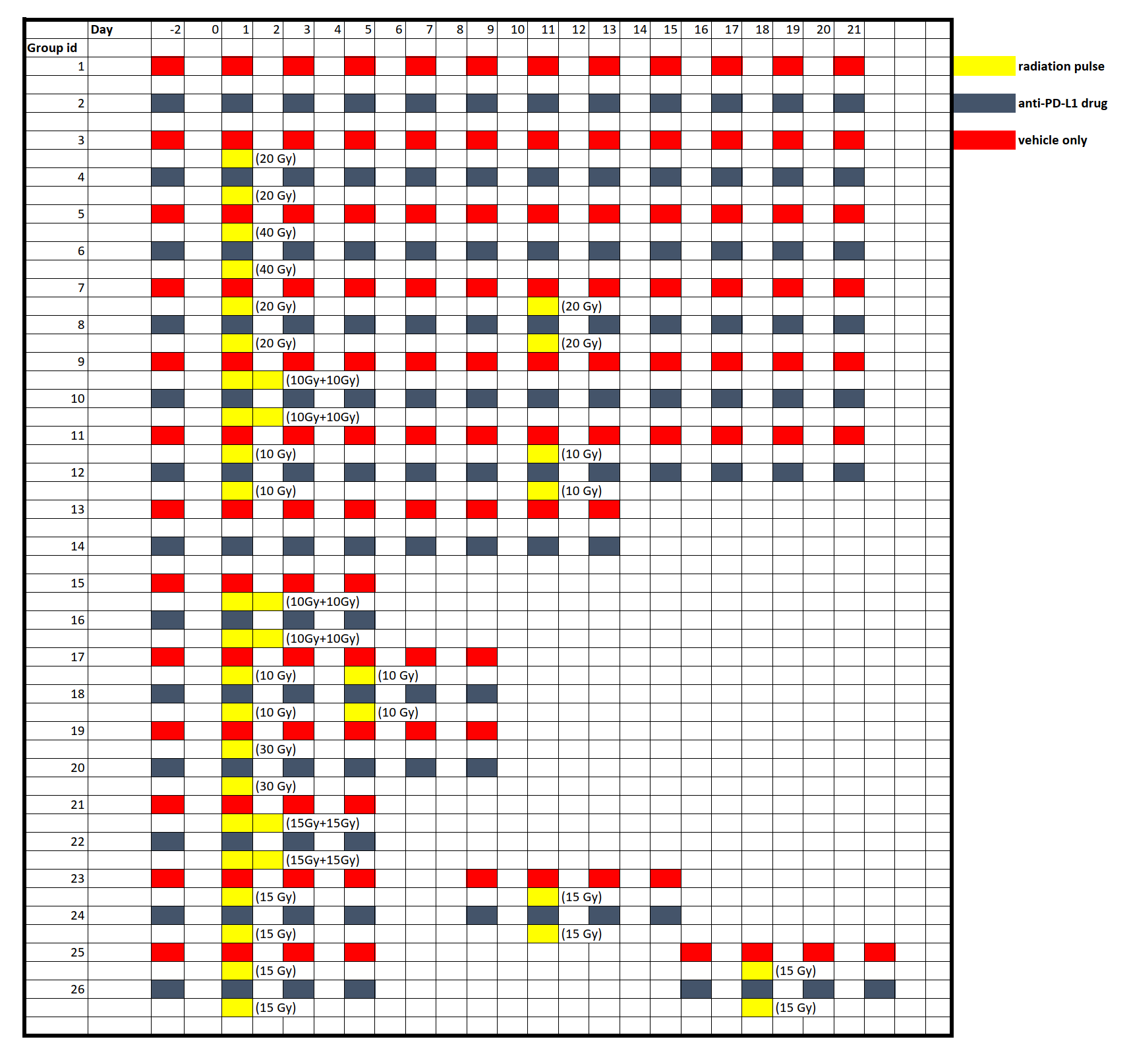

Supplement: Supplementary file 1 [file DataSheet1.docx]
